# CTD: Fast, accurate, and interpretable method for static and dynamic tensor decompositions

**DOI:** 10.1371/journal.pone.0200579

**Published:** 2018-07-25

**Authors:** Jungwoo Lee, Dongjin Choi, Lee Sael

**Affiliations:** Department of Computer Science and Engineering, Seoul National University, Seoul, Republic of Korea; Univerisity of Oxford, UNITED KINGDOM

## Abstract

How can we find patterns and anomalies in a tensor, i.e., multi-dimensional array, in an efficient and directly interpretable way? How can we do this in an online environment, where a new tensor arrives at each time step? Finding patterns and anomalies in multi-dimensional data have many important applications, including building safety monitoring, health monitoring, cyber security, terrorist detection, and fake user detection in social networks. Standard tensor decomposition results are not directly interpretable and few methods that propose to increase interpretability need to be made faster, more memory efficient, and more accurate for large and quickly generated data in the online environment. We propose two versions of a fast, accurate, and directly interpretable tensor decomposition method we call CTD that is based on efficient sampling method. First is the static version of CTD, i.e., CTD-S, that provably guarantees up to 11× higher accuracy than that of the state-of-the-art method. Also, CTD-S is made up to 2.3× faster and up to 24× more memory-efficient than the state-of-the-art method by removing redundancy. Second is the dynamic version of CTD, i.e. CTD-D, which is the first interpretable dynamic tensor decomposition method ever proposed. It is also made up to 82× faster than the already fast CTD-S by exploiting factors at previous time step and by reordering operations. With CTD, we demonstrate how the results can be effectively interpreted in online distributed denial of service (DDoS) attack detection and online troll detection.

## Introduction

Given a tensor, or multi-dimensional array, how can we find patterns and anomalies in an efficient and directly interpretable way? How can we do this in an online environment, where new data arrive at each time step? Many real-world data are multi-dimensional and can be modeled as sparse tensors. Examples include network traffic data (source IP—destination IP—time), movie rating data (user—movie—time), IoT sensor data, and healthcare data. Finding patterns and anomalies in those tensor data is a very important problem with many applications such as building safety monitoring [1], patient health monitoring [2–5], cyber security [6], terrorist detection [7–9], and fake user detection in social networks [10, 11]. Tensor decomposition method, a widely-used tool in tensor analysis, has been used for this task. However, the standard tensor decomposition methods such as PARAFAC [12] and Tucker [13] do not provide interpretability and are not applicable for real-time analysis in environments with high-velocity data.

Sampling-based tensor decomposition methods [14–16] arose as an alternative due to their direct interpretability. The direct interpretability not only reduces time and effort involved in finding patterns and anomalies from the decomposed tensors but also provides clarity in interpreting the result. A sampling-based decomposition method for sparse tensors is also memory-efficient since it preserves the sparsity of the original tensors on the sampled factor matrices. However, existing sampling-based tensor decomposition methods are slow, have high memory usage, and produce low accuracy. For example, Tensor-CUR [16], the state-of-the-art sampling-based static tensor decomposition method, has many redundant fibers including duplicates in its factors. These redundancy cause higher memory usage and longer running time. Tensor-CUR is also not accurate enough for real-world tensor analysis.

In addition to interpretability, demands for online method applicable in a dynamic environment, where multi-dimensional data are generated continuously at a fast rate, are also increasing. A real-time analysis is not feasible with static methods since all the data, i.e., historical and incoming tensors, need to be decomposed over again at each time step. There are a few dynamic tensor decomposition methods proposed [17–19]. However, proposed methods are not directly interpretable and do not preserve sparsity. To the best of our knowledge, there has been no sampling-based dynamic tensor decomposition method proposed.

In this paper, we propose CTD (Compact Tensor Decomposition), a fast, accurate, and interpretable sampling-based tensor decomposition method. CTD has two versions: CTD-S for static tensors, and CTD-D for dynamic tensors. CTD-S is optimal after sampling, and results in a compact tensor decomposition through careful sampling and redundancy elimination, thereby providing much better running time and memory efficiency than previous methods. CTD-D, the first sampling-based dynamic tensor decomposition method in literature, updates and modifies minimally on the components altered by the incoming data, making the method applicable for real-time analysis on a dynamic environment. Table 1 shows the comparison of CTD and the existing method, Tensor-CUR.

**Table 1:** Comparison of our proposed CTD and the existing Tensor-CUR. The static method CTD-S outperforms the state of-the-art Tensor-CUR in terms of time, memory usage, and accuracy. The dynamic method CTD-D is the fastest.

	Existing	[Proposed]	
	Tensor-CUR [16]	CTD-S	CTD-D
**Interpretability**	✓	✓	✓
**Time**	fast	faster	**fastest**
**Memory usage**	low	**lower**	low
**Accuracy**	low	**high**	**high**
**Online**	⨯	⨯	✓

Our main contributions are as follows:

- **Method.** We propose CTD, a fast, accurate, and directly interpretable tensor decomposition method. We prove the optimality of the static method CTD-S which makes it more accurate than the state-of-the-art method. Also, to the best of our knowledge, the dynamic method CTD-D is the first sampling-based dynamic tensor decomposition method.
- **Performance.** CTD-S is up to 11× more accurate, 2.3× faster, and 24× more memory-efficient compared to Tensor-CUR, the state-of-the-art competitor. CTD-D is up to 82× faster than CTD-S.
- **Interpretable Analysis.** We show how CTD results are directly interpreted to successfully detect DDoS attacks in network traffic data and trolls in social network data.

The codes and datasets used in this paper are available at https://github.com/leesael/CTD. The rest of this paper is organized as follows. We first describe preliminaries and related works for tensor and sampling-based decomposition. We then describe our proposed method CTD and the experimental results. After presenting CTD at work, we conclude this paper.

## Preliminaries and related works

In this section, we describe preliminaries and related works for tensor and sampling-based decompositions. Table 2 lists the definitions of symbols used in this paper.

**Table 2:** Table of symbols.

Symbol	Definition	Symbol	Definition
X	tensor (Euler script, bold letter)	**X**^†^	pseudoinverse of **X**
**X**	matrix (uppercase, bold letter)	*N*	order of a tensor
**x**	column vector (lower case, bold letter)	×_*n*_	*n*-mode product
*x*	scalar (lower case, italic letter)	‖•‖_*F*_	Frobenius norm
**X**_(*n*)_	mode-*n* matricization of a tensor X	nnz(X)	number of nonzero elements in X

### Tensor

A tensor is a multi-dimensional array and is denoted by the boldface Euler script, e.g. $X\in R^{I_{1}\times \cdots \times I_{N}}$ where *N* denotes the order (the number of axes) of X. Each axis of a tensor is also known as mode or way. A fiber is a vector (1-mode tensor) which has fixed indices except one. Every index of a mode-*n* fiber is fixed except *n*-th index. A fiber can be regarded as a higher-order version of a matrix row and column. A matrix column and row each correspond to mode-1 fiber and mode-2 fiber, respectively. A slab is an (*N* − 1)-mode tensor which has only one fixed index. $X_{\left(\alpha \right)}\in R^{I_{\alpha }\times N_{\alpha }}$ denotes a mode-*α* matricization of X, where *N*_*α*_ = ∏_*n* ≠ *α*_ *I*_*n*_. **X**_(*α*)_ is made by rearranging mode-*α* fibers of X to be the columns of **X**_(*α*)_. ${∥X∥}_{F}$ is the Frobenius norm of X and is defined by Eq (1).

$$\begin{array}{c} {∥X∥}_{F}^{2}=\sum_{i_{1},i_{2},\cdots ,i_{N}}x_{i_{1}i_{2}\cdots i_{N}}^{2} \end{array}$$ (1)

$X\times_{n}U\in R^{I_{1}\times \cdots \times I_{n-1}\times J\times I_{n+1}\times \cdots \times I_{N}}$ denotes the *n*-mode product of a tensor $X\in R^{I_{1}\times \cdots \times I_{N}}$ with a matrix $U\in R^{J\times I_{n}}$. Elementwise,

$$\begin{array}{c} {\left(X\times_{n}U\right)}_{i_{1}\cdots i_{n-1}ji_{n+1}\cdots i_{N}}=\sum_{i_{n}=1}^{I_{n}}x_{i_{1}\cdots i_{n-1}i_{n}i_{n+1}\cdots i_{N}}u_{ji_{n}} \end{array}$$ (2)

$X\times_{n}U$ has a property shown in Eq (3).

$$\begin{array}{c} Y=X\times_{n}U\Leftrightarrow Y_{\left(n\right)}=UX_{\left(n\right)} \end{array}$$ (3)

We assume that a matrix or tensor is stored in a sparse-unordered representation (i.e. only nonzero entries are stored in a form of pair of indices and the corresponding value). nnz(X) denotes the number of nonzero elements in X.

We describe existing sampling-based matrix and tensor decomposition methods in the following subsections.

### Sampling based matrix decomposition

Sampling-based matrix decomposition methods sample columns or rows from a given matrix and use them to make their factors. They produce directly interpretable factors which preserve sparsity since those factors directly reflect the sparsity of the original data. In contrast, a singular value decomposition (SVD) generates factors which are hard to understand and dense because the factors are in a form of linear combination of columns or rows from the given matrix. Definition 1 shows the definition for CX matrix decomposition [20], a kind of sampling-based matrix decomposition.

**Definition 1.** *Given a matrix* $A\in R^{m\times n}$, *the matrix* $\tilde{A}=CX$ *is a CX matrix decomposition of* **A**, *where a matrix* $C\in R^{m\times c}$ *consists of actual columns of* **A** *and a matrix* **X** *is any matrix of size* *c* × *n*.

We introduce well-known CX matrix decomposition methods: LinearTimeCUR, CMD, and Colibri.

#### LinearTimeCUR and CMD

Drineas et al. [21] proposed LinearTimeCUR and Sun et al. [22] proposed CMD. In the initial step, LinearTimeCUR and CMD sample columns from an original matrix **A** according to the probabilities proportional to the norm of each column with replacement. Drineas et al. [21] has proven that this biased sampling provides an optimal approximation. Then, they project **A** into the column space spanned by those sampled columns and use the projection as the low-rank approximation of **A**. LinearTimeCUR has many duplicates in its factors because a column or row with a higher norm is likely to be selected multiple times. These duplicates make LinearTimeCUR slow and require a large amount of memory. CMD handles the duplication issue by removing duplicate columns and rows in the factors of LinearTimeCUR, thereby reducing running time and memory significantly.

#### Colibri

Tong et al. [23] proposed Colibri-S which improves CMD by removing all types of linear dependencies including duplicates. Colibri-S is much faster and memory-efficient compared to LinearTimeCUR and CMD because the dimension of factors is much smaller than that of LinearTimeCUR and CMD. Tong et al. [23] also proposed the dynamic version Colibri-D. Although Colibri-D can update its factors incrementally, it fixes the indices of the initially sampled columns which need to be updated over time. Our CTD-D not only handles general dynamic tensors but also does not have to fix those indices.

### Sampling based tensor decomposition

Sampling-based tensor decomposition method samples actual fibers or slabs from an original tensor. In contrast to PARAFAC [12] and Tucker [13], the most famous tensor decomposition methods, the resulting factors of sampling-based tensor decomposition method are easy to understand and usually sparse. There are two types of sampling based tensor decomposition: one based on Tucker and the other based on LR tensor decomposition which is defined in Definition 2.2. In Tucker-type sampling based tensor decomposition (e.g., ApproxTensorSVD [14] and FBTD (fiber-based tensor decomposition) [15]), factor matrices for all modes are either sampled or generated; the overhead of generating a factor matrix for each mode makes these methods too slow for applications to real-time analysis. We focus on sampling methods based on LR tensor decomposition which is faster than those based on Tucker decomposition.

**Definition 2.** (*LR tensor decomposition*) *Given a tensor* $X\in R^{I_{1}\times I_{2}\times \cdots \times I_{N}}$, $\tilde{X}=L\times_{\alpha }R$ *is a mode*-*α* *LR tensor decomposition of* X, *where a matrix* $R\in R^{I_{\alpha }\times c}$ *consists of actual mode*-*α* *fibers of* X *and a tensor* L *is any tensor of size* *I*_1_ × ⋯ × *I*_*α*−1_ × *c* × *I*_*α*+1_ × ⋯ × *I*_*N*_.

Tensor-CUR. Mahoney et al. [16] proposed Tensor-CUR, a mode-*α* LR tensor decomposition method. Tensor-CUR is an *n*-dimensional extension of LinearTimeCUR. Tensor-CUR samples fibers and slabs from an original tensor and builds its factors using the sampled ones. The only difference between LinearTimeCUR and Tensor-CUR is that Tensor-CUR exploits fibers and slabs instead of columns and rows. Thus, Tensor-CUR has drawbacks similar to those of LinearTimeCUR. Tensor-CUR has many redundant fibers in its factors and these fibers make Tensor-CUR slow and use a large amount of memory.

## Proposed method

In this section, we describe our proposed CTD (Compact Tensor Decomposition), an efficient and interpretable sampling-based tensor decomposition method. We first describe the static version CTD-S, and then the dynamic version CTD-D of CTD.

### CTD-S for static tensors

#### Overview

How can we design an efficient sampling-based static tensor decomposition method? Tensor-CUR, the existing state-of-the-art, has many redundant fibers in its factors and these fibers make Tensor-CUR slow and use large memory. Our proposed CTD-S method removes all dependencies from the sampled fibers and maintains only independent fibers; thus, CTD-S is faster and more memory-efficient than Tensor-CUR.

#### Algorithm

**Fig 1:**
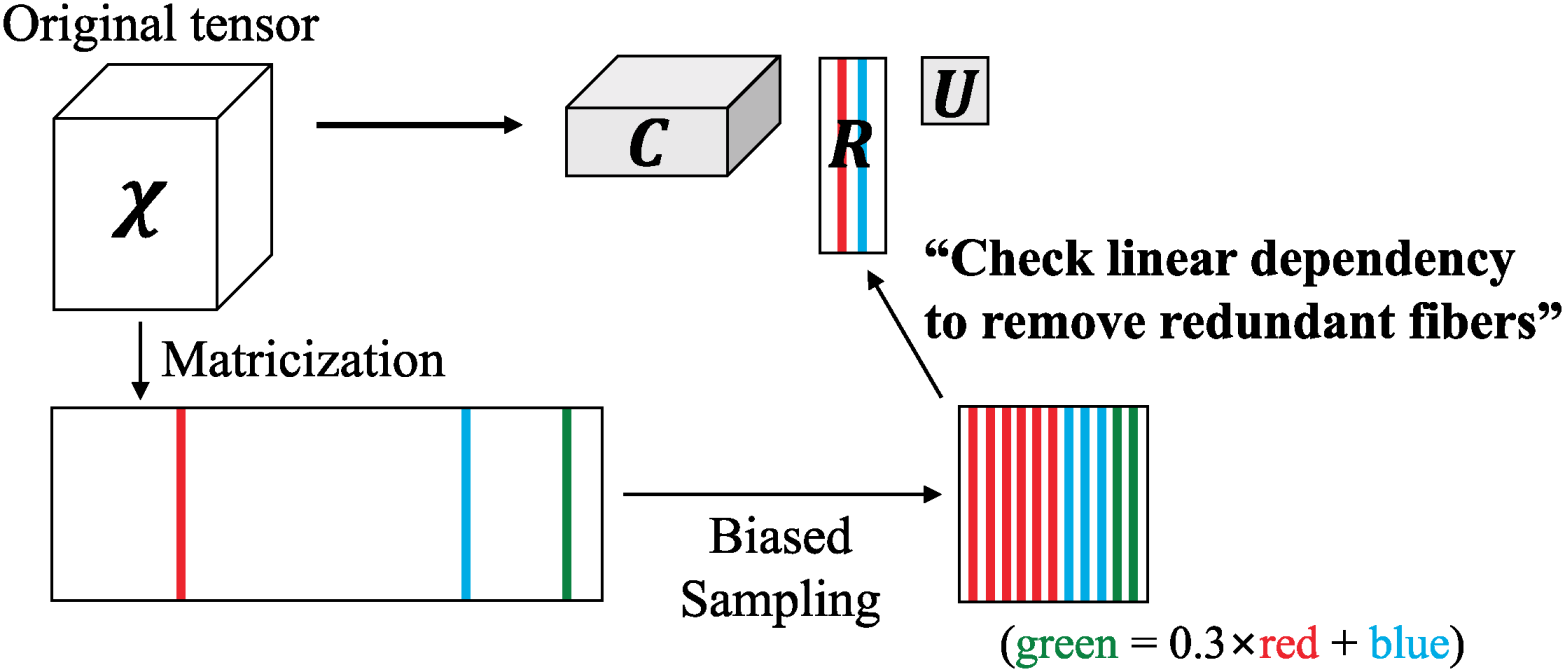
The scheme for CTD-S.

Fig 1 shows the scheme for CTD-S. CTD-S first samples fibers biased toward a norm of each fiber. Three different fibers (red, blue, green) are sampled in Fig 1. There are many duplicates after biased sampling process since CTD-S samples fibers multiple times with replacement and a fiber with a higher norm is likely to be sampled many times. There also exist linearly dependent fibers such as the green fiber which can be expressed as a linear combination of the red one and the blue one. Those linearly dependent fibers including duplicates are redundant in that they do not give new information when interpreting the result. CTD-S removes those redundant fibers and stores only the independent fibers in its factor **R** to keep result compact. CTD-S only keeps one red fiber and one blue fiber in **R** in Fig 1.

CTD-S decomposes a tensor $X\in R^{I_{1}\times I_{2}\times \cdots \times I_{N}}$ into one tensor $C\in R^{I_{1}\times \cdots \times I_{\alpha -1}\times \tilde{s}\times I_{\alpha +1}\times \cdots \times I_{N}}$, and two matrices $U\in R^{\tilde{s}\times \tilde{s}}$ and $R\in R^{I_{\alpha }\times \tilde{s}}$ such that $X\approx C\times_{\alpha }RU$. CTD-S is a mode-*α* LR tensor decomposition method and is interpretable since **R** consists of independent fibers sampled from X.

**Algorithm 1** CTD-S for Static Tensor

**Input:** Tensor $X\in R^{I_{1}\times I_{2}\times \cdots \times I_{N}}$, mode *α* ∈ {1, ⋯, *N*}, sample size *s* ∈ {1, ⋯, *N*_*α*_}, and tolerance *ϵ*

**Output:** $C\in R^{I_{1}\times \cdots \times I_{\alpha -1}\times \tilde{s}\times I_{\alpha +1}\times \cdots \times I_{N}}$, $U\in R^{\tilde{s}\times \tilde{s}}$, $R\in R^{I_{\alpha }\times \tilde{s}}$

1: Let **X**_(*α*)_ be the mode-*α* matricization of X

2: Compute column distribution for *i* = 1, ⋯, *N*_*α*_: $P\left(i\right)\leftarrow \frac{|X_{\left(\alpha \right)}{\left(:,i\right)|}^{2}}{∥X_{\left(\alpha \right)}{∥}_{F}^{2}}$

3: Sample *s* columns from **X**_(*α*)_ based on *P*(*i*). Let *I* = {*i*_1_, ⋯, *i*_*s*_}

4: Let $I^{\prime }=\left\{i_{1}^{\prime },\cdots ,i_{s^{\prime }}^{\prime }\right\}$ be a set consisting of unique elements in *I*

5: Initialize $R\leftarrow [X_{\left(\alpha \right)}\left(:,i_{1}^{\prime }\right)]$ and $U\leftarrow 1/(X_{\left(\alpha \right)}{\left(:,i_{1}^{\prime }\right)}^{T}X_{\left(\alpha \right)}\left(:,i_{1}^{\prime }\right))$

6: **for** *k* = 2: *s*′ **do**

7:  Compute the residual:

  $\vec{res}\leftarrow \left(X_{\left(\alpha \right)}\left(:,i_{k}^{\prime }\right)-{\text{RUR}}^{T}X_{\left(\alpha \right)}\left(:,i_{k}^{\prime }\right)\right)$

8:  **if** $\left|\right|\vec{res}\left|\right|\leq \epsilon ||X_{\left(\alpha \right)}\left(:,i_{k}^{\prime }\right)\left|\right|$ **then**

9:   continue

10: **else**

11:   Compute: $\delta \leftarrow \left|\right|\vec{res}{\left|\right|}^{2}$ and $\vec{y}\leftarrow UR^{T}X_{\left(\alpha \right)}\left(:,i_{k}^{\prime }\right)$

12:   Update **U**: $U\leftarrow (\begin{array}{cc} U+\vec{y}{\vec{y}}^{T}/\delta & -\vec{y}/\delta \\ -{\vec{y}}^{T}/\delta & 1/\delta \end{array})$

13:   Expand $R:R\leftarrow [R,X_{\left(\alpha \right)}\left(:,i_{k}^{\prime }\right)]$

14:  **end if**

15: **end for**

16: Compute $C\leftarrow X\times_{\alpha }R^{T}$

17: **return** C, **U**, **R**

Algorithm 1 shows the procedure of CTD-S. First, CTD-S computes the probabilities of mode-*α* fibers of X, which are proportional to the norm of each fiber, and then samples *s* fibers from X according to the probabilities with replacement, in lines 1-3. Redundant fibers exist in the sampled fibers in this step. CTD-S selects unique fibers from the initially sampled *s* fibers in line 4 where *s*′ denotes the number of those unique fibers. This step reduces the number of iterations in lines 6-15 from *s* − 1 to *s*′ − 1. **R** is initialized by the first sampled fiber in line 5. In lines 6-15, CTD-S removes redundant mode-*α* fibers in the sampled fibers. The matrices **U** and **R** are computed incrementally in this step. The columns of **R** always consist of independent mode-*α* fibers through the loop. In each iteration, CTD-S checks whether one of the sampled fibers is linearly independent of the column space spanned by **R** or not in lines 7-8, using the residual tolerance *ϵ*. If the fiber is independent, CTD-S updates **U** and expands **R** with the fiber in lines 10-13. Finally, CTD-S computes C with X and **R** in line 16.

Lemma 1 shows the computational cost of CTD-S.

**Lemma 1.** *The computational complexity of CTD-S is* $O(\left(\tilde{s}I_{\alpha }+s\right)N_{\alpha }+s^{\prime }\left({\tilde{s}}^{2}+nnz\left(R\right)\right)+s\log s+nnz\left(X\right))$, *where* *N*_*α*_ *is* ∏_*n* ≠ *α*_ *I*_*n*_ *and* $\tilde{s}⪡s^{\prime }\leq s$.

*Proof.* The mode-*α* matricization of X in line 1 needs O(nnz(X)) operations. Computing column distribution in line 2 takes $O(nnz\left(X\right)+N_{\alpha })$ and sampling *s* columns in line 3 takes $O(sN_{\alpha })$. O(slogs) operation is required in computing unique elements in *I* in line 4. Computing **R** and **U** in lines 5-15 takes $O(s^{\prime }\left({\tilde{s}}^{2}+nnz\left(R\right)\right))$ as proved in Lemma 1 in [23]. Computing C in line 16 takes $O(\tilde{s}I_{\alpha }N_{\alpha })$. Overall, CTD-S needs $O(\left(\tilde{s}I_{\alpha }+s\right)N_{\alpha }+s^{\prime }\left({\tilde{s}}^{2}+nnz\left(R\right)\right)+s\log s+nnz\left(X\right))$ operations.

Lemma 2 shows that CTD-S has the optimal accuracy for given sampled fibers and *ϵ* = 0, thus is more accurate than Tensor-CUR.

**Lemma 2.** *CTD-S has the minimum error, thus is more accurate than* Tensor-CUR *for a given* **R**_0_ *consisting of initially sampled fibers when the residual tolerance* *ϵ* = 0.

*Proof.* CTD-S and Tensor-CUR are both mode-*α* LR tensor decomposition methods. They both sample fibers from X in the same way in the initial step. Assume **R**_0_ be the matrix consisting of those initially sampled fibers, and the same **R**_0_ is given for CTD-S and Tensor-CUR. Then, the reconstruction error of X given **R**_0_ is a function of **L**_(*α*)_ as shown in Eq (4). The equality comes from Eq (3).

$$\begin{array}{c} ||X-L\times_{\alpha }R_{0}{\left|\right|}_{F}=\left|\right|X_{\left(\alpha \right)}-R_{0}L_{\left(\alpha \right)}{\left|\right|}_{F} \end{array}$$ (4)

The reconstruction error is globally minimum when $L_{\left(\alpha \right)}=R_{0}^{†}X_{\left(\alpha \right)}$. Eq (5) shows the minimum reconstruction error.

$$\begin{array}{c} \min_{L_{\left(\alpha \right)}}\left|\right|X_{\left(\alpha \right)}-R_{0}L_{\left(\alpha \right)}{\left|\right|}_{F}=\left|\right|X_{\left(\alpha \right)}-R_{0}R_{0}^{†}X_{\left(\alpha \right)}{\left|\right|}_{F} \end{array}$$ (5)

Let **R** be the factor of CTD-S. **R** consists of the independent columns of **R**_0_ since the tolerance *ϵ* = 0. We show that CTD-S has the minimum reconstruction error in Eq (6).

$$\begin{array}{ccc} \left|\right|X_{\left(\alpha \right)}-R_{0}R_{0}^{†}X_{\left(\alpha \right)}{\left|\right|}_{F} & = & \left|\right|X_{\left(\alpha \right)}-RR^{†}X_{\left(\alpha \right)}{\left|\right|}_{F}\\ & = & \left|\right|X_{\left(\alpha \right)}-R{\left(R^{T}R\right)}^{-1}R^{T}X_{\left(\alpha \right)}{\left|\right|}_{F}\\ & = & \left|\right|X_{\left(\alpha \right)}-RUC_{\left(\alpha \right)}{\left|\right|}_{F}\\ & = & \;\text{Error}\;\text{of}\;\text{CTD-S} \end{array}$$ (6)

The first equality in Eq (6) holds because $R_{0}R_{0}^{†}X_{\left(\alpha \right)}$ means the projection of **X**_(*α*)_ onto the column space of **R**_0_, and **R** and **R**_0_ have the same column space. The third equality holds because CTD-S uses (**R**^*T*^ **R**)^−1^ for its factor **U** (theorem 1 in [23]), and **R**^*T*^ **X**_(*α*)_ for its factor C. In contrast, Tensor-CUR does not have the minimum reconstruction error because Tensor-CUR has **L**_(*α*)_ which is different from $R_{0}^{†}X_{\left(\alpha \right)}$. Specifically, Tensor-CUR further samples rows (called slabs) from **X**_(*α*)_ to construct its **L**_(*α*)_.

### CTD-D for dynamic tensors

#### Overview

How can we design an efficient sampling-based dynamic tensor decomposition method? In a dynamic setting, a new tensor arrives at every time step and we want to keep track of sampling-based tensor decomposition. The main challenge is to update factors quickly while preserving accuracy. Note that there has been no sampling-based dynamic tensor decomposition method in the literature. Applying CTD-S at every time step is not a feasible option since it starts from scratch to update its factors, and thus running time increases rapidly as tensor grows. We propose CTD-D, the first sampling-based dynamic tensor decomposition method. CTD-D samples mode-*α* fibers only from the newly arrived tensor, and then updates the factors appropriately using those sampled ones. The main idea of CTD-D is to update the factors of CTD-S incrementally by (1) exploiting factors at previous time step and (2) reordering operations.

**Algorithm 2** CTD-D for Dynamic Tensor

**Input:** Tensor $\Delta X\in R^{I_{1}\times \cdots \times I_{N-1}\times 1}$, mode *α* ∈ {1, ⋯, *N* − 1}, $C^{\left(t\right)}$, **U**^(*t*)^, **R**^(*t*)^, sample size *d* ∈ {1, ⋯, Δ*N*_*α*_}, and tolerance *ϵ*

**Output:** $C^{\left(t+1\right)}$, **U**^(*t*+1)^, **R**^(*t*+1)^

1: Let Δ**X**_(*α*)_ be the mode-*α* matricization of ΔX

2: Compute column distribution for *i* = 1, ⋯, Δ*N*_*α*_:

$P\left(i\right)\leftarrow \frac{|\Delta X_{\left(\alpha \right)}{\left(:,i\right)|}^{2}}{∥\Delta X_{\left(\alpha \right)}{∥}_{F}^{2}}$

3: Sample *d* columns from Δ**X**_(*α*)_ based on *P*(*i*). Let *I* = {*i*_1_, ⋯, *i*_*d*_}

4: Let $I^{\prime }=\left\{i_{1}^{\prime },\cdots ,i_{d^{\prime }}^{\prime }\right\}$ be a set consisting of unique elements in *I*

5: Initialize **R**^(*t*+1)^ ← **R**^(*t*)^, **U**^(*t*+1)^ ← **U**^(*t*)^, and Δ**R** ← []

6: **for** *k* = 1: *d*′ **do**

7:  Let $x\leftarrow \Delta X_{\left(\alpha \right)}\left(:,i_{k}^{\prime }\right)$

8:  Compute the residual:

  $\vec{res}\leftarrow \left(x-R^{\left(t+1\right)}U^{\left(t+1\right)}{\left(R^{\left(t+1\right)}\right)}^{T}x\right)$

9:  **if** $\left|\right|\vec{res}\left|\right|\leq \epsilon ||x||$ **then**

10:   continue

11:  **else**

12:   Compute: $\delta \leftarrow \left|\right|\vec{res}{\left|\right|}^{2}$ and $\vec{y}\leftarrow U^{\left(t+1\right)}{\left(R^{\left(t+1\right)}\right)}^{T}x$

13:   Update **U**^(*t*+1)^: $U^{\left(t+1\right)}\leftarrow (\begin{array}{cc} U^{\left(t+1\right)}+\vec{y}{\vec{y}}^{T}/\delta & -\vec{y}/\delta \\ -{\vec{y}}^{T}/\delta & 1/\delta \end{array})$

14:   Expand **R**^(*t*+1)^ and Δ**R**: **R**^(*t*+1)^ ← [**R**^(*t*+1)^,**x**] and Δ**R** ← [Δ**R**,**x**]

15:  **end if**

16: **end for**

  Update $C_{\left(\alpha \right)}^{\left(t+1\right)}$:

17: **if** Δ**R** is not empty **then**

18:  $C_{\left(\alpha \right)}^{\left(t+1\right)}\leftarrow (\begin{array}{cc} C_{\left(\alpha \right)}^{\left(t\right)} & {\left(R^{\left(t\right)}\right)}^{T}\Delta X_{\left(\alpha \right)}\\ {\left(\Delta R\right)}^{T}R^{\left(t\right)}U^{\left(t\right)}C_{\left(\alpha \right)}^{\left(t\right)} & {\left(\Delta R\right)}^{T}\Delta X_{\left(\alpha \right)} \end{array})$

19: **else**

20:  $C_{\left(\alpha \right)}^{\left(t+1\right)}\leftarrow (\begin{array}{cc} C_{\left(\alpha \right)}^{\left(t\right)} & {\left(R^{\left(t\right)}\right)}^{T}\Delta X_{\left(\alpha \right)} \end{array})$

21: **end if**

22: Fold $C_{\left(\alpha \right)}^{\left(t+1\right)}$ into $C^{\left(t+1\right)}$

23: **return** $C^{\left(t+1\right)}$, **U**^(*t*+1)^, **R**^(*t*+1)^

#### Algorithm

**Fig 2:**
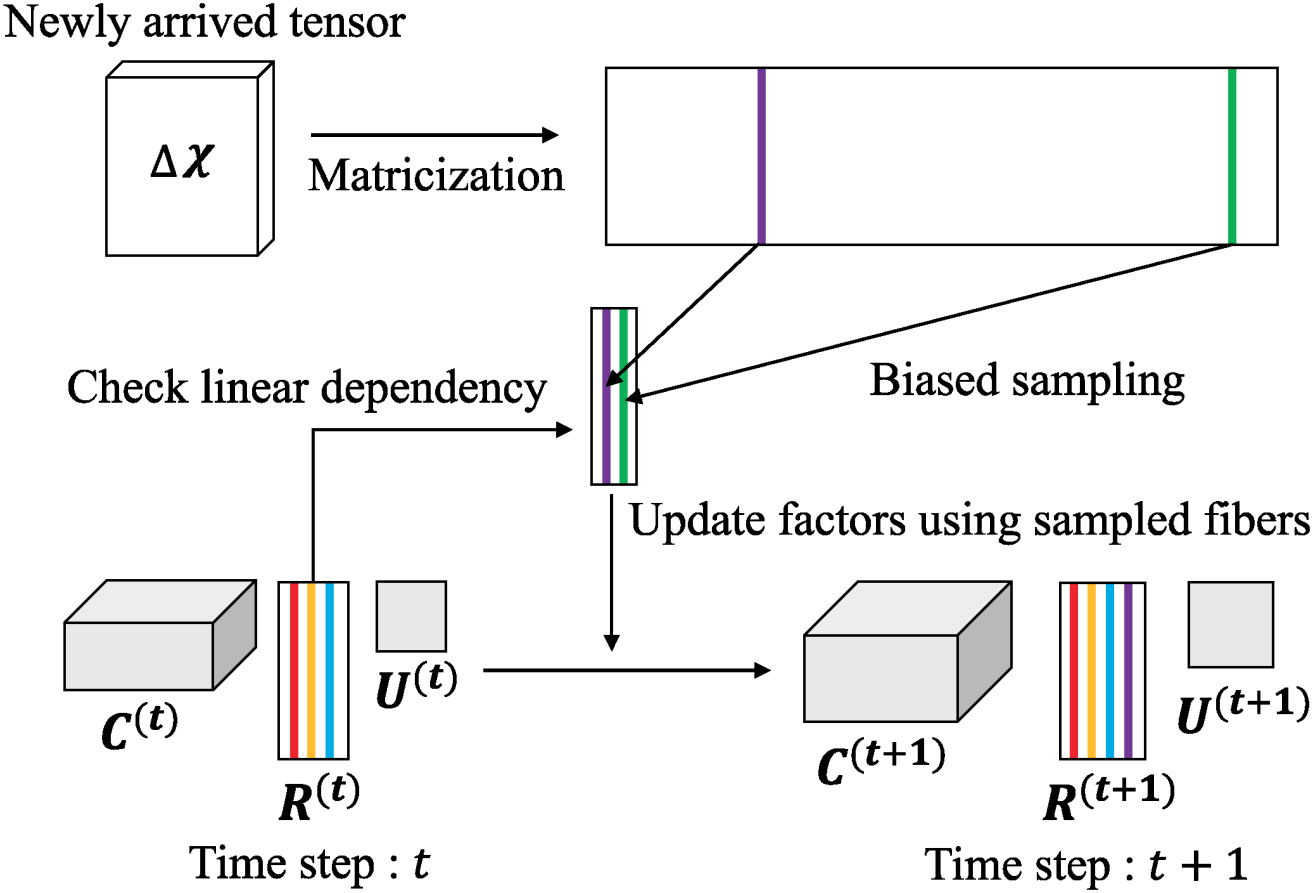
The scheme for CTD-D.

Fig 2 shows the scheme for CTD-D. At each time step, CTD-D samples fibers from newly arrived tensor and updates factors by checking linear dependency of sampled fibers with the factor at previous time step. Purple and green fiber are sampled from newly arrived tensor in Fig 2. Note that the purple fiber is added to the factor **R** since it is linearly independent of the fibers in the factor at the previous time step, while the linearly dependent green fiber is ignored.

For any time step *t*, CTD-D maintains its factors $C^{\left(t\right)}\in R^{I_{1}\times \cdots \times I_{\alpha -1}\times {\tilde{d}}_{t}\times I_{\alpha +1}\times \cdots \times I_{N-1}\times t}$, $U^{\left(t\right)}\in R^{{\tilde{d}}_{t}\times {\tilde{d}}_{t}}$, and $R^{\left(t\right)}\in R^{I_{\alpha }\times {\tilde{d}}_{t}}$ such that $X^{\left(t\right)}\approx C^{\left(t\right)}\times_{\alpha }R^{\left(t\right)}U^{\left(t\right)}$, where the upper subscript (*t*) indicates that the factor is at time step *t*. $X^{\left(t\right)}$ grows along the time mode and we assume that *N*-th mode is the time mode in a dynamic setting, where *N* denotes the order of $X^{\left(t\right)}$. At the next time step *t* + 1, CTD-D receives newly arrived tensor $\Delta X\in R^{I_{1}\times I_{2}\times \cdots \times \cdots \times I_{N-1}\times 1}$ and updates $C^{\left(t\right)}$, **U**^(*t*)^, and **R**^(*t*)^ into $C^{\left(t+1\right)}\in R^{I_{1}\times \cdots \times I_{\alpha -1}\times {\tilde{d}}_{t+1}\times I_{\alpha +1}\times \cdots \times I_{N-1}\times \left(t+1\right)}$, $U^{\left(t+1\right)}\in R^{{\tilde{d}}_{t+1}\times {\tilde{d}}_{t+1}}$, and $R^{\left(t+1\right)}\in R^{I_{\alpha }\times {\tilde{d}}_{t+1}}$, respectively such that $X^{\left(t+1\right)}\approx C^{\left(t+1\right)}\times_{\alpha }R^{\left(t+1\right)}U^{\left(t+1\right)}$.

Algorithm 2 shows the procedure of CTD-D. First, CTD-D computes the probabilities of mode-*α* fibers of ΔX, which are proportional to the norm of each fiber, and then samples *d* fibers according to the probabilities with replacement in lines 1-3. CTD-D selects unique *d*′ fibers in line 4 and initializes **R**^(*t*+1)^, **U**^(*t*+1)^, and Δ**R** with **R**^(*t*)^, **U**^(*t*)^, and an empty matrix respectively in line 5, where Δ**R** consists of differences between **R**^(*t*)^ and **R**^(*t*+1)^. In lines 6-16, CTD-D expands **R**^(*t*+1)^ with those sampled fibers by sequentially evaluating linear dependency of each fiber with the column space of **R**^(*t*+1)^. **R**^(*t*+1)^ and **U**^(*t*+1)^ are updated in this step. Finally, $C_{\left(\alpha \right)}^{\left(t+1\right)}$ is updated in lines 17-21.

In the following, we describe two main ideas of CTD-D to update $C_{\left(\alpha \right)}^{\left(t+1\right)}$, **R**^(*t*+1)^, and **U**^(*t*+1)^ efficiently while preserving accuracy: exploiting factors at previous time step, and reordering operations.

**(1) Exploiting factors at previous time step:** First, we explain how we update **R**^(*t*+1)^ and **U**^(*t*+1)^ using the idea. In line 5 of Algorithm 1, CTD-S initializes **R** and **U** using one of the sampled fibers. This is because CTD-S requires **R** to consist of linearly independent columns and it is satisfied when **R** has only one fiber. Since **R**^(*t*)^ already consists of linearly independent columns, we initialize **R**^(*t*+1)^ and **U**^(*t*+1)^ with **R**^(*t*)^ and **U**^(*t*)^ respectively in line 5 of Algorithm 2. In lines 6-16, we check linear independence of each sampled fiber from Δ**X**_(*α*)_ with **R**^(*t*+1)^. If the fiber is linearly independent, we expand **R**^(*t*+1)^ and update **U**^(*t*+1)^ as in the lines 11-13 of Algorithm 1.

Second, we describe how we update $C^{\left(t+1\right)}$ using the idea. We assume that Δ**R** is not empty after line 16 of Algorithm 2. At time step *t* and its successor step *t* + 1, CTD-S satisfies Eqs (7) and (8), where $C_{\left(\alpha \right)}^{\left(t\right)}$ has the size ${\tilde{d}}_{t}\times N_{\alpha }^{\left(t\right)}$ and $C_{\left(\alpha \right)}^{\left(t+1\right)}$ has the size ${\tilde{d}}_{t+1}\times N_{\alpha }^{\left(t+1\right)}$.

$$\begin{array}{c} C_{\left(\alpha \right)}^{\left(t\right)}\leftarrow {\left(R^{\left(t\right)}\right)}^{T}X_{\left(\alpha \right)}^{\left(t\right)} \end{array}$$ (7)

$$\begin{array}{c} C_{\left(\alpha \right)}^{\left(t+1\right)}\leftarrow {\left(R^{\left(t+1\right)}\right)}^{T}X_{\left(\alpha \right)}^{\left(t+1\right)} \end{array}$$ (8)

We can rewrite **R**^(*t*+1)^ and $X_{\left(\alpha \right)}^{\left(t+1\right)}$ as Eqs (9) and (10) respectively, where Δ**R** has the size $I_{\alpha }\times \Delta \tilde{d}$ and Δ**X**_(*α*)_ has the size *I*_*α*_×Δ*N*_*α*_ such that $N_{\alpha }^{\left(t+1\right)}=N_{\alpha }^{\left(t\right)}+\Delta N_{\alpha }$ and ${\tilde{d}}_{t+1}={\tilde{d}}_{t}+\Delta \tilde{d}$.

$$\begin{array}{c} R^{\left(t+1\right)}=\left[\begin{array}{cc} R^{\left(t\right)} & \Delta R \end{array}\right] \end{array}$$ (9)

$$\begin{array}{c} X_{\left(\alpha \right)}^{\left(t+1\right)}=\left[\begin{array}{cc} X_{\left(\alpha \right)}^{\left(t\right)} & \Delta X_{\left(\alpha \right)} \end{array}\right] \end{array}$$ (10)

We replace **R**^(*t*+1)^ and $X_{\left(\alpha \right)}^{\left(t+1\right)}$ in Eq (8) with those in Eqs (9) and (10), respectively, to obtain the Eq (11).

$$\begin{array}{ccc} C_{\left(\alpha \right)}^{\left(t+1\right)} & \leftarrow & \left[\begin{array}{c} {\left(R^{\left(t\right)}\right)}^{T}\\ {\left(\Delta R\right)}^{T} \end{array}\right]\left[\begin{array}{cc} X_{\left(\alpha \right)}^{\left(t\right)} & \Delta X_{\left(\alpha \right)} \end{array}\right]\\ & = & \left[\begin{array}{cc} {\left(R^{\left(t\right)}\right)}^{T}X_{\left(\alpha \right)}^{\left(t\right)} & {\left(R^{\left(t\right)}\right)}^{T}\Delta X_{\left(\alpha \right)}\\ {\left(\Delta R\right)}^{T}X_{\left(\alpha \right)}^{\left(t\right)} & {\left(\Delta R\right)}^{T}\Delta X_{\left(\alpha \right)} \end{array}\right] \end{array}$$ (11)

CTD-S computes all the 4 elements (${\left(R^{\left(t\right)}\right)}^{T}X_{\left(\alpha \right)}^{\left(t\right)}$, (**R**^(*t*)^)^*T*^Δ**X**_(*α*)_, ${\left(\Delta R\right)}^{T}X_{\left(\alpha \right)}^{\left(t\right)}$, and (Δ**R**)^*T*^Δ**X**_(*α*)_) in Eq (11) from scratch, hence requires a lot of computations. To make computation of $C_{\left(\alpha \right)}^{\left(t+1\right)}$ incremental, we exploit existing factors at time step *t*: $C_{\left(\alpha \right)}^{\left(t\right)}$, **R**^(*t*)^, and **U**^(*t*)^. First, we use $C_{\left(\alpha \right)}^{\left(t\right)}$ instead of ${\left(R^{\left(t\right)}\right)}^{T}X_{\left(\alpha \right)}^{\left(t\right)}$ as in the Eq (7). Second, we should replace $X_{\left(\alpha \right)}^{\left(t\right)}$ in ${\left(\Delta R\right)}^{T}X_{\left(\alpha \right)}^{\left(t\right)}$ with the factors at time step *t*, since CTD-D does not have $X_{\left(\alpha \right)}^{\left(t\right)}$ as its input unlike CTD-S. We substitute $R^{\left(t\right)}U^{\left(t\right)}C_{\left(\alpha \right)}^{\left(t\right)}$ for $X_{\left(\alpha \right)}^{\left(t\right)}$. This is because CTD-S ensures $X^{\left(t\right)}\approx C^{\left(t\right)}\times_{\alpha }R^{\left(t\right)}U^{\left(t\right)}$ which can be rewritten as $X_{\left(\alpha \right)}^{\left(t\right)}\approx R^{\left(t\right)}U^{\left(t\right)}C_{\left(\alpha \right)}^{\left(t\right)}$ by Eq (3). Eq (12) shows the final form of $C_{\left(\alpha \right)}^{\left(t+1\right)}$ which is the same as line 18 in Algorithm 2.

$$\begin{array}{c} C_{\left(\alpha \right)}^{\left(t+1\right)}\leftarrow \left[\begin{array}{cc} C_{\left(\alpha \right)}^{\left(t\right)} & {\left(R^{\left(t\right)}\right)}^{T}\Delta X_{\left(\alpha \right)}\\ {\left(\Delta R\right)}^{T}R^{\left(t\right)}U^{\left(t\right)}C_{\left(\alpha \right)}^{\left(t\right)} & {\left(\Delta R\right)}^{T}\Delta X_{\left(\alpha \right)} \end{array}\right] \end{array}$$ (12)

${\left(\Delta R\right)}^{T}R^{\left(t\right)}U^{\left(t\right)}C_{\left(\alpha \right)}^{\left(t\right)}$ and (Δ**R**)^*T*^Δ**X**_(*α*)_ are ignored when Δ**R** is empty as expressed in line 20 of Algorithm 2.

**(2) Reordering computations:** The computation order for the element ${\left(\Delta R\right)}^{T}R^{\left(t\right)}U^{\left(t\right)}C_{\left(\alpha \right)}^{\left(t\right)}$ is important since each order has a different computation cost. We want to determine the optimal parenthesization among possible parenthesizations. It can be shown that $\left(\left({\left(\Delta R\right)}^{T}R^{\left(t\right)}\right)U^{\left(t\right)}\right)C_{\left(\alpha \right)}^{\left(t\right)}$ is the optimal one with $O(\left(\Delta \tilde{d}\right){\tilde{d}}_{t}\left(I_{\alpha }+{\tilde{d}}_{t}+N_{\alpha }^{\left(t\right)}\right))$ operations and can be done by parenthesizing from the left.

We prove that CTD-D is faster than CTD-S in Lemma 3.

**Lemma 3.** *CTD-D is faster than CTD-S. The computational complexity of CTD-D is* $O(\left(\Delta \tilde{d}\right){\tilde{d}}_{t}\left(N_{\alpha }^{\left(t\right)}+I_{\alpha }\right)+\left({\tilde{d}}_{t+1}I_{\alpha }+d\right)\Delta N_{\alpha }+d^{\prime }\left({\tilde{d}}_{t+1}^{2}+nnz\left(R^{\left(t+1\right)}\right)\right)+d\log d+nnz\left(\Delta X\right))$.

*Proof.* The lines 1-4 of Algorithm 2 for CTD-D are similar to those of Algorithm 1 for CTD-S. The only difference is that CTD-D samples *d* columns from Δ**X**_(*α*)_ while CTD-S samples *s* columns from **X**_(*α*)_. Thus, lines 1-4 takes $O(nnz\left(\Delta X\right)+d\Delta N_{\alpha }+d\log d)$. Updating **R**^(*t*+1)^ and **U**^(*t*+1)^ in lines 5-16 needs $O(d^{\prime }\left({\tilde{d}}_{t+1}^{2}+nnz\left(R^{\left(t+1\right)}\right)\right))$ operations as proved in Lemma 1 in [23]. In updating $C^{\left(t+1\right)}$ in lines 17-18, (**R**^(*t*)^)^*T*^Δ**X**_(*α*)_ takes computational cost of $O({\tilde{d}}_{t}I_{\alpha }\Delta N_{\alpha })$. (Δ**R**)^*T*^Δ**X**_(*α*)_ takes $O(\Delta \tilde{d}I_{\alpha }\Delta N_{\alpha })$ and ${\left(\Delta R\right)}^{T}R^{\left(t\right)}U^{\left(t\right)}C_{\left(\alpha \right)}^{\left(t\right)}$ takes $O(\left(\Delta \tilde{d}\right){\tilde{d}}_{t}\left(I_{\alpha }+{\tilde{d}}_{t}+N_{\alpha }^{\left(t\right)}\right))$. Overall, CTD-D takes $O(\left(\Delta \tilde{d}\right){\tilde{d}}_{t}\left(N_{\alpha }^{\left(t\right)}+I_{\alpha }\right)+\left({\tilde{d}}_{t+1}I_{\alpha }+d\right)\Delta N_{\alpha }+d^{\prime }\left({\tilde{d}}_{t+1}^{2}+nnz\left(R^{\left(t+1\right)}\right)\right)+d\log d+nnz\left(\Delta X\right))$.

CTD-D is faster than CTD-S because CTD-S has $\tilde{s}I_{\alpha }N_{\alpha }$ in its complexity, which is much larger than all the terms in the complexity of CTD-D.

## Experiments

We perform experiments to answer the following questions.

**Q1**: What is the performance of our static method CTD-S compared to the competing method Tensor-CUR?

**Q2**: How do the performance of CTD-S and Tensor-CUR change with regard to the sample size parameter?

**Q3**: What is the performance of our dynamic method CTD-D compared to the static method CTD-S?

**Q4**: What are the results of applying CTD-D for online DDoS attack detection and online troll detection?

### Experimental settings

#### Machine

All the experiments are performed on a machine with a 10-core Intel 2.20 GHz CPU and 256 GB RAM.

#### Competing method

We compare our proposed method CTD with Tensor-CUR [16], the state-of-the-art sampling-based tensor decomposition method. Both methods are implemented in MATLAB.

#### Measure

We define three metrics (1. *Relative Error*, 2. *Memory*, and 3. *Time*) as follows. First, a *Relative Error* is defined as Eq (13). X denotes the original tensor and $\tilde{X}$ is the tensor reconstructed from the factors of X. For example, $\tilde{X}=C\times_{\alpha }RU$ in CTD-S.

$$\begin{array}{c} Relative\;Error=\frac{\left|\right|\tilde{X}-X{\left|\right|}_{F}^{2}}{{\left||X|\right|}_{F}^{2}} \end{array}$$ (13)

Second, *Memory* is defined as Eq (14). It measures the relative amount of memory needed for storing the resulting factors. The denominator and numerator indicate the amount of memory needed for storing the original tensor and the resulting factors, respectively.

$$\begin{array}{c} Memory=\frac{nnz(C)+nnz(U)+nnz(R)}{nnz(X)} \end{array}$$ (14)

Finally, *Time* denotes running time in seconds.

#### Data

Table 3 shows the data we used in our experiments.

**Table 3:** Summary of the tensor data used.

Name	*I*_1_	*I*_2_	*I*_3_	Nonzeros
Facebook-wall [25]	63,891	63,890	1,504	738,485
Facebook-wall (synthetic) [30]	63,891	63,890	1,504	1,169,656
Hyperspectral Image [26]	538	323	148	25,715,854
Infectious [27]	407	410	1,392	17,298
Hypertext 2009 [28]	112	113	5,246	20,818
Haggle [29]	77	274	1,567	27,972
CAIDA [30]	189	189	1,000	20,511
CAIDA (synthetic) [30]	189	189	1,000	46,102

#### Input parameters

All methods take a tensor X generated from each dataset, a mode *α*, and a sample size *s* as input because they are LR tensor decomposition methods. In each experiment, we give the same input and compare the performance. We fix *α* = 1 and perform experiments under various sample sizes *s*. We set the number of slabs to sample *r* = *s* and the rank *k* = 10 in Tensor-CUR, and set *ϵ* = 10^−6^ in CTD.

### Performance of CTD-S

We measure the performance of CTD-S to answer Q1 and Q2. In summary, compared to the Tensor-CUR, CTD-S is more accurate, and its running time and memory usage are relatively constant over various sample sizes.

**Fig 3:**
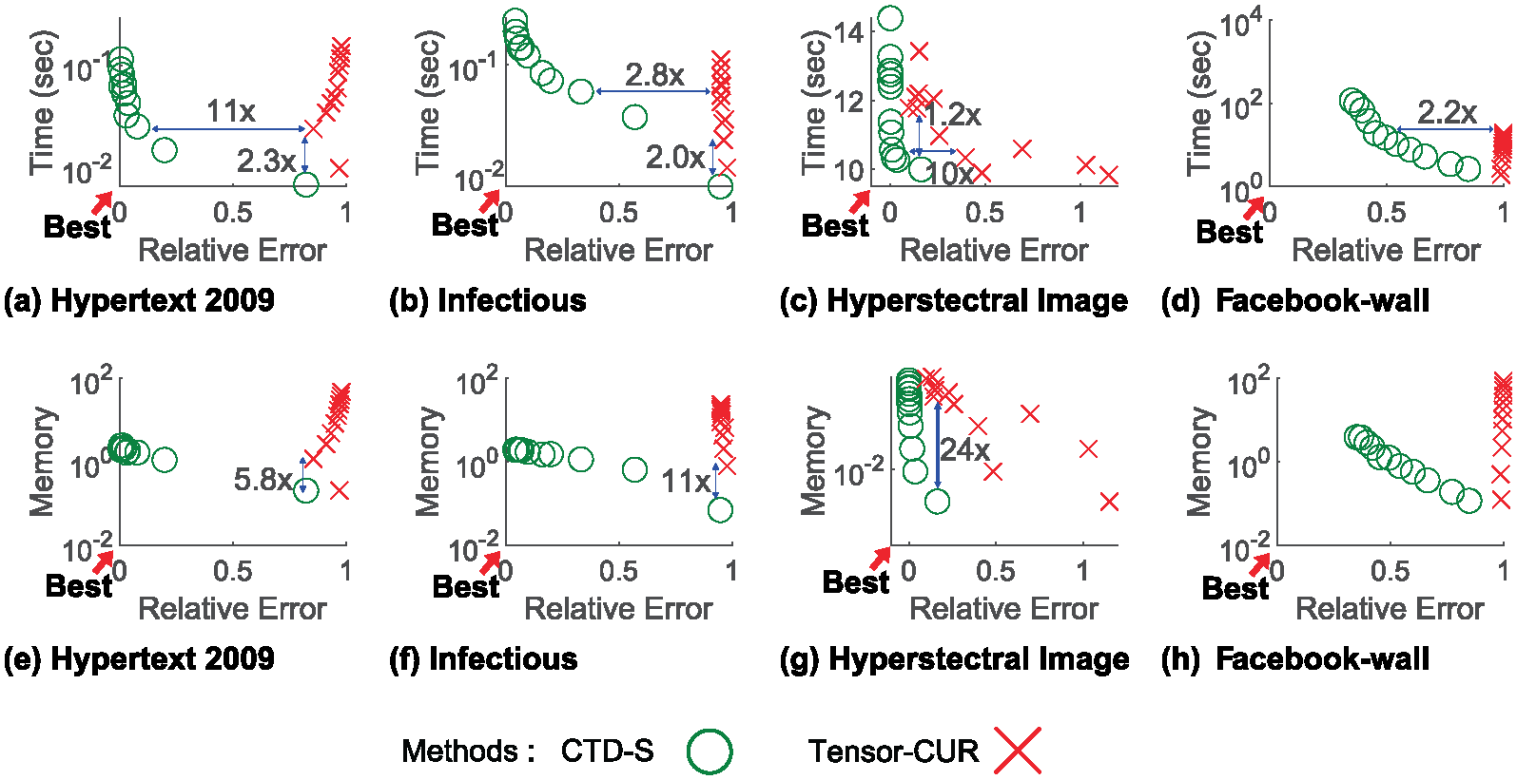
Error, running time, and memory usage of CTD-S compared to Tensor-CUR varying sample sizes. CTD-S is more accurate, faster and more memory-efficient than Tensor-CUR.

Fig 3 shows the *Time* vs. *Relative Error* and the *Memory* vs. *Relative Error* of CTD-S compared to Tensor-CUR under various sample sizes to answer Q1. We measure error under similar level of running time with the pair of results with smallest difference in running time (horizontal lines in Fig 3). We find that CTD-S is up to 11× more accurate for the same level of running time compared to Tensor-CUR. This phenomenon coincides with the Lemma 2, which guarantees that CTD-S is more accurate than Tensor-CUR theoretically. Likewise, we choose the pair of points with smallest error difference between the two methods to compare running time and memory (vertical lines in Fig 3). Although no significant improvement in speed and memory usages are found for under similar sample sizes, CTD-S is able to perform 2.3× faster, and 24× more memory-efficient than Tensor-CUR under similar error rates.

**Fig 4:**
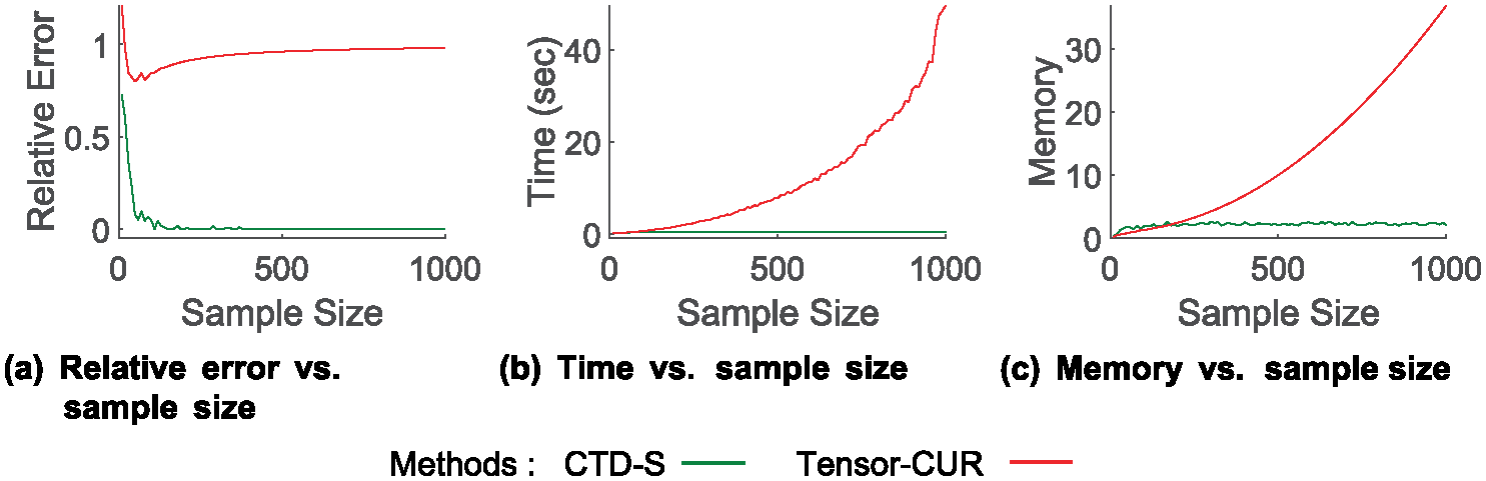
Error, running time, and memory usage of CTD-S compared to those of Tensor-CUR over sample size *s* for haggle dataset. CTD-S is more accurate over various sample sizes, and its running time and memory usage are relatively constant compared to the Tensor-CUR.

Fig 4 shows the *Relative Error*, *Time*, and *Memory* of CTD-S compared to those of Tensor-CUR over increasing sample sizes *s* for the Haggle dataset to answer question Q2. The error of CTD-S decreases as *s* increases because it gains more data to sample important fibers which describe the original tensor well. The running time and memory usage of CTD-S are relatively constant compared to those of Tensor-CUR. This is because CTD-S keeps only the linearly independent fibers, the number of which is bound by the rank of **X**_(*α*)_. There are small fluctuations in the graphs since the sampling process of both CTD-S and Tensor-CUR are based on randomness. Although we have shown the results for only the Haggle dataset, the overall trend persists over other datasets.

We further investigate the differences in accuracy improvements under various datasets by characterizing the dataset and relations to CTD-S performs. we characterize the datasets with density (dense or sparse) and fiber independence rate. The fiber independence rates are measured as follows:

$$\begin{array}{c} \frac{\text{number}\;\text{of}\;\text{independent}\;\text{mode-}\alpha \;\text{fibers}}{\text{number}\;\text{of}\;\text{the}\;\text{whole}\;\text{mode-}\alpha \text{fibers}\;\text{of}\;\text{a}\;\text{tensor}} \end{array}$$

Table 4 represents the accuracy of CTD-S compared to Tensor-CUR and fiber independence rate. We can identify that in sparse datasets, ones with a lower fiber independence rate shows better accuracy performance in large. This is in an accordance to the assumption that if the fiber independence rate is low, there’s a high probability of obtaining most of these independent fibers with a given sample size leading to high accuracy for CTD-S. The Hyperspectral Image data that forms a dense tensor showed relatively higher accuracy even though the proportion of independent fibers is high under the strict measure of independence. However, image data are known to have high redundancy and CTD-S samples fibers well even when strict independence rate is low.

**Table 4:** The accuracy of CTD-S compared to Tensor-CUR and the fiber independence rate.

Name	Density	Accuracy compared to Tensor-CUR	Fiber independence rate
CAIDA	sparse	48.3×	3.81 × 10^−6^
Haggle	sparse	30.1×	1.24 × 10^−6^
Hypertext 2009	sparse	11×	1.79 × 10^−4^
Facebook-wall	sparse	2.2×	4.40 × 10^−4^
Infectious	sparse	2.8×	6.43 × 10^−4^
Hyperspectral Image	dense	10×	1.13 × 10^−2^

### Performance of CTD-D

We compare the performance of CTD-D with those of CTD-S to answer Q3. In summary, CTD-D is up to 82× faster for the same level of error compared to CTD-S. The detail is as follows.

To simulate a dynamic environment, we divide a given dataset into two parts along the time mode. We use the first 80% of the dataset as historical data and the later 20% as incoming data. We assume that historical data is already given and incoming data arrives sequentially at every time step, such that the whole data grows along the time mode. We measure the performance of CTD-D and CTD-S at each time step and calculate the average. We set the sample size *d* of CTD-D to be much smaller than that of CTD-S because CTD-D samples fibers only from the increment ΔX while CTD-S samples from the whole data X. We set *d* of CTD-D to be 0.01 times *s* of CTD-S, *α* = 1, and *ϵ* = 10^−6^.

**Fig 5:**
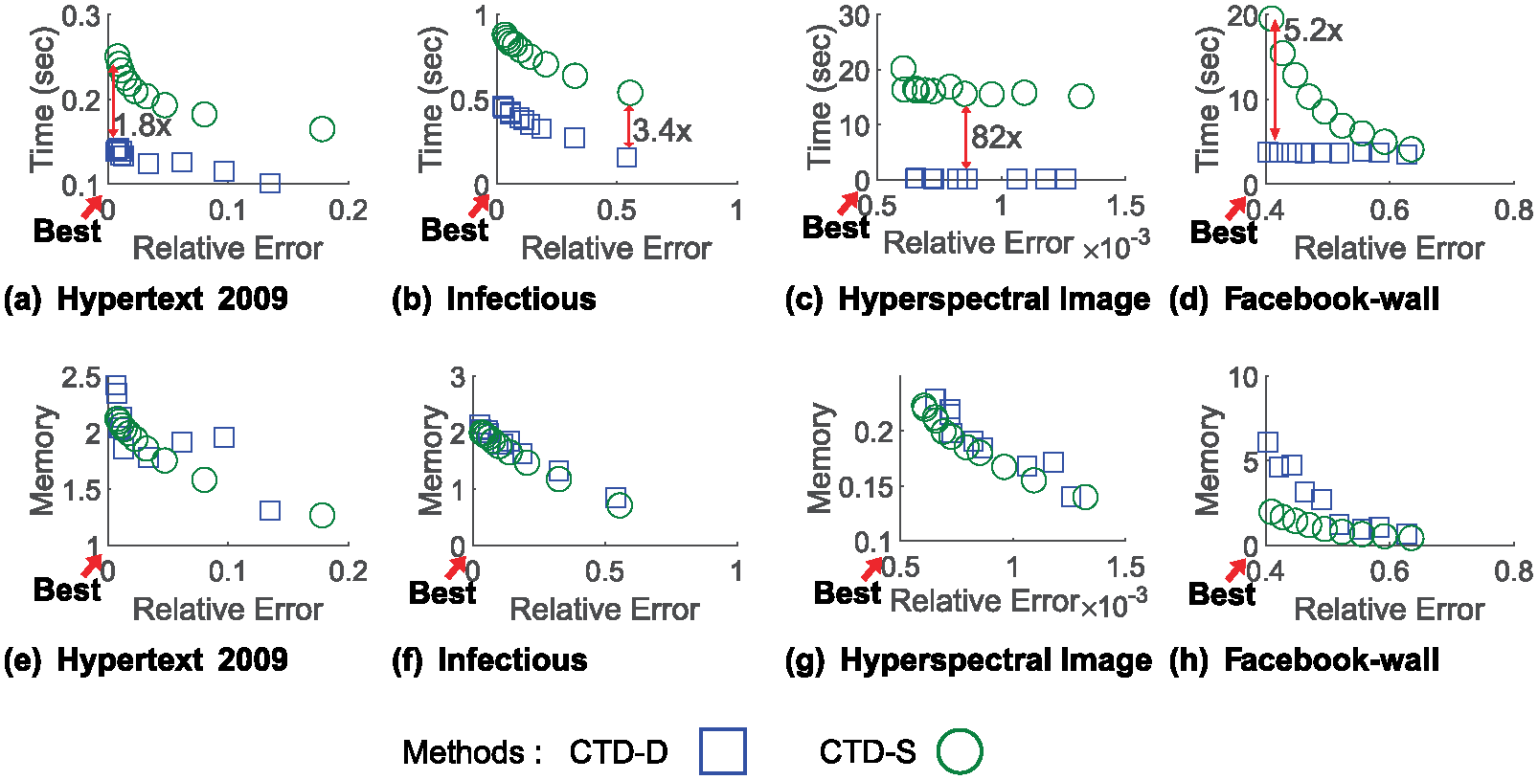
Error, running time, and memory usage relation of CTD-D compared CTD-S varying sample sizes. CTD-D is faster and has smaller error while using the same or slightly larger memory space compared to CTD-S.

Fig 5 shows the *Time* vs. *Relative Error* and *Memory* vs. *Relative Error* relation of CTD-D compared to those of CTD-S. Note that CTD-D is much faster than CTD-S for all the datasets. The reason why CTD-D is especially faster for the Hyperspectral Image dataset is that the dataset has relatively many dependent fibers, which makes CTD-D skip updating **U**, compared to the other datasets. CTD-D uses the same or slightly more memory than CTD-S does. This is because multiplication between sparse matrices used in updating C does not always produce sparse output, thus the number of nonzero entries in C increases slightly over time steps.

## CTD at work

In this section, we apply CTD-D to online DDoS attack detection in network traffic data and online troll detection in social network data. We show how CTD-D’s interpretability can help successfully detect DDoS attacks and trolls.

### Online DDoS attack detection

A DDoS attack makes an online service unavailable by sending a huge amount of traffic to the server from multiple sources. DDoS attacks are still major threats to many companies. In effect, 20% of financial companies get $1 million revenue loss per hour and 43% lose more than $250,000 hourly under DDoS attack, while 74% take more than 1 hour to shut down the attacks [24].

Our goal is to detect DDoS attacks in network traffic data efficiently in an online fashion. We propose a novel online DDoS attack detection method based on CTD-D’s interpretability. We show that CTD-D is one of the feasible options for online DDoS attack detection and show how it detects attacks successfully. In contrast to the standard PARAFAC [12] and Tucker [13] decomposition methods, CTD-D can determine DDoS attacks from its decomposition result without expensive overhead. We aim to dynamically find a victim (destination host) and corresponding attackers (source hosts) of each DDOS attack in network traffic data that is when a victim receives a huge amount of traffic from a large number of attackers.

The online DDoS attack detection method based on CTD-D is as follows. First, we apply CTD-D on network traffic data which is a 3-mode tensor in the form of (source IP—destination IP—time). We assume an online environment where each slab of the network traffic data in the form of (source IP—destination IP) arrives sequentially at every time step. We use source IP mode as mode *α*. Second, we inspect the factor **R** of CTD-D, which consists of actual mode-*α* fibers from the original data. **R** is composed of important mode-*α* fibers which signify major activities such as DDoS attack or heavy traffic to the main server. Thanks to CTD, we can directly find out destination host and occurrence time of a major activity represented in a fiber in **R**, by simply tracking the indices of fibers. We regard fibers with the same destination host index represent the same major activity, and consider the first fiber among those with the same destination host index to be the representative of each major activity. Then, we select fibers with the norm higher than the average among the first fibers and suggest them as candidates of DDoS attack. This is because DDoS attacks have much higher norms than normal traffic does.

We generate network traffic data by injecting DDoS attacks on the real-world CAIDA network traffic dataset [30]. We assume that randomly selected 20% of source hosts participate in each DDoS attack. Table 5 shows the result of DDoS attack detection method of CTD-D. CTD-D achieves high F1 score for various number *n* of injected DDoS attacks with notable precision. We set *d* = 10, and *ϵ* = 0.15.

**Table 5:** The result of online DDoS attack detection method based on CTD-D. CTD-D achieves high F1 score for various *n* with notable precision, where *n* denotes the number of injected DDoS attacks.

n	Recall	Precision	F1 score
1	1.000	1.000	1.000
3	1.000	1.000	1.000
5	0.880	1.000	0.931
7	0.857	1.000	0.921

### Online troll detection

Recent social network services (SNS) such as Facebook or Twitter has billions of users; their main concern is to detect trolls, or abnormal users, since trolls can severely undermine the service. Our goal is to detect trolls in social network tensor data in an online fashion. We define a troll as an abnormal user who posts on the other users’ walls much more than normal users do. We show how CTD-D finds trolls successfully using its interpretability.

We use a process similar to the online DDoS attack detection method based on CTD-D described in the previous section to find trolls. We use the real-world Facebook-wall social network tensor, a 3-mode tensor containing triplets where each entry denotes the number of posts for the corresponding triplet. A triplet (User 1—User 2—time) means that User 2 posted on the User 1’s wall. We assume an online environment where new data point in the form of (User 1—User 2) arrives at every time step. We apply CTD-D with User 1 mode for *α* so that each fiber collected in the factor **R** represents User 2’s behavior at some time. By tracking indices of fibers in the factor **R**, we can reveal which fiber represents behaviors of which users at which time. We then decide trolls (User 2) by picking fibers which have norm larger than the average.

We test the ability of CTD to interpretability detect trolls by inserting synthetic trolls into the Facebook-wall dataset. Table 6 shows the result of online troll detection in Facebook-wall dataset based on CTD-D. It is notable that we can detect all the trolls inserted with very small sample size, 10^−4^% of the entire fibers, for a various number of trolls.

**Table 6:** The result of online troll detection in facebook-wall dataset based on CTD-D. CTD-D detects all the trolls inserted (*recall* = 1) for various *n*, where *n* denotes the number of injected troll users. Note that we used only 10^−4^% of the entire fibers as a sample size.

n	Recall	Precision	F1 score
1	1.000	0.200	0.333
3	1.000	0.500	0.667
5	1.000	0.556	0.714
10	1.000	0.833	0.909

## Conclusion

We propose CTD, a fast, accurate, and directly interpretable tensor decomposition method based on sampling. The static version CTD-S is up to 11× more accurate, 2.3× faster, and 24× more memory-efficient compared to the state-of-the-art method. The dynamic version CTD-D is up to 82× faster than CTD-S for an online environment. CTD-D is the first method providing interpretable *dynamic* tensor decomposition. Utilizing the interpretability of CTD, we were able to successfully detect online DDoS attacks and trolls from network data. The interpretability of CTD comes from the assumption that the original data fiber itself is sparse and interpretable, such as IP address or words in documents. Although not all real-world data have this property, such as values in gene expression data, a wide range of social and technological data in the online environment does have the sparse and interpretable properties. In such cases, CTD is capable of dynamically detecting important or abnormal data in an online environment.
